# Characterization of Nasopharyngeal Microbiota Dysbiosis in Children with *Mycoplasma pneumoniae* Pneumonia

**DOI:** 10.3390/microorganisms14061374

**Published:** 2026-06-21

**Authors:** Jing Bi, Bo Yu, Yang Zhang, Guotong Zheng, Yiyuan Han, Yangyan Yan, Wen Wang, Lei Wu, Yingshuo Wang, Zhengkai Yi

**Affiliations:** 1 Department of ENT and Head & Neck Surgery, The Children’s Hospital, Zhejiang University School of Medicine, Hangzhou 310051, China; bijing-ent@zju.edu.cn (J.B.); yubo-ent@zju.edu.cn (B.Y.); 6516022@zju.edu.cn (Y.Z.); 6522004@zju.edu.cn (G.Z.); noe308744@163.com (Y.H.); 6513043@zju.edu.cn (Y.Y.); 2 College of Life Sciences, China Jiliang University, Hangzhou 310018, China; wangwen@cjlu.edu.cn; 3 Key Laboratory of Microbiological Metrology, Measurement & Bio-Product Quality Security, State Administration for Market Regulation, China Jiliang University, Hangzhou 310018, China; 4 Department of Respiratory Medicine, The Children’s Hospital, Zhejiang University School of Medicine, Hangzhou 310051, China; zjuent@zju.edu.cn

**Keywords:** *Mycoplasma pneumoniae* pneumonia, nasopharyngeal microbiota, 16S rRNA, children

## Abstract

*Mycoplasma pneumoniae* pneumonia (MPP) is a leading cause of community-acquired pneumonia in children, yet little is known about the role of nasopharyngeal microbiota dysbiosis in susceptibility to infection and disease subtype. In this study, we performed 16S rRNA sequencing on nasopharyngeal samples from 102 pediatric MPP patients, 104 influenza A patients, and 103 healthy controls and compared the microbial diversity, composition, and functional profiles across groups. The MPP group exhibits an altered nasopharyngeal microbial composition, characterized by reduced microbial diversity and an increased relative abundance of genera including *Mycoplasma*, *Pseudomonas*, *Acinetobacter*, and *Tannerella*. Distinct microbiota profiles were identified for the MPP subtypes, with *Mycoplasma* more abundant in bronchopneumonia (BP) than in lobar pneumonia (LP). A microbial classifier based on the relative abundance of the nasopharyngeal microbiota was established to distinguish MPP patients from both influenza patients and healthy controls, with an area under the receiver operating characteristic curves of 0.978. Key microbial features associated with MPP included *Mycoplasma*, *Mycobacterium*, *Aeromonas*, and *Acinetobacter*. In addition, PICRUSt2-based functional predictions suggested alterations in amino acid metabolism and predicted functional pathways associated with bacterial infection and antimicrobial resistance in MPP patients. In conclusion, this study provides comprehensive insights into alterations in the nasopharyngeal microbiota in pediatric MPP. These findings highlight the potential role of dysbiosis in disease progression and suggest that changes in microbiota composition and functional profiles are associated with MPP infection.

## 1. Introduction

*Mycoplasma pneumoniae* (MP) is the smallest self-replicating prokaryote without a cell wall. It possesses a compact and highly stable genome with a size of approximately 800 kb and is a major causative agent of acute respiratory tract infections, accounting for 18.6% of reported cases [1]. *Mycoplasma pneumoniae* pneumonia (MPP), primarily transmitted through respiratory droplets, contributes to 20–40% of community-acquired pneumonia (CAP) cases in children [2]. Although most infections are mild and self-limiting, some cases may progress to severe or critical conditions accompanied by life-threatening extrapulmonary complications [3].

The respiratory tract microbiota, which is typically balanced in healthy individuals, plays a critical role in maintaining host defenses and preventing pathogen colonization [4,5]. The disruption of this balance by pathogens, environmental changes, or immune disturbances can lead to microbial dysbiosis and symptomatic infections, including pneumonia [6,7]. Emerging evidence links changes in respiratory microbiota with acute respiratory infections (ARIs), such as bronchiolitis and pneumonia [4,8]. MPP, a common lower respiratory tract infection (LRTI) in children, is associated with dysbiosis in the lower respiratory tract microbiota [9,10]. Recent studies have explored the role of microbiota in the development of MPP, highlighting potential associations with the gut, lung, nasopharyngeal (NP), or oropharyngeal (OP) microbiota [9,11,12]. The upper respiratory tract (URT), particularly the nasopharynx with its high microbial density, serves as a key microenvironment in respiratory infections [9,13]. The nasopharynx is not only representative of the airway microbiota but is also a significant source for lung microbiota through microbial exchange. Previous research has highlighted changes in the NP and OP microbiota during ARIs, indicating potential microbial transmission between these regions [10,14]. However, few studies specifically examine the role of the URT microbiota in MPP progression in children, with the existing research often being limited by small sample sizes or a non-pediatric focus [4,9,10]. Therefore, a comprehensive analysis of the nasopharyngeal microbiota in children with MPP is essential for understanding the alterations in the microenvironment and associated functional gene changes.

This study aims to assess URT microbiota dysbiosis in children with MPP and explore its association with disease types. Using 16S ribosomal RNA (rRNA) sequencing, we analyzed the nasopharyngeal microbiota of 102 children with MPP, comparing them with 104 influenza A patients and 103 healthy controls. We evaluated microbial diversity, the bacterial relative abundance, and functional gene profiles to characterize the distinct microbiota landscape in MPP and its correlation with disease subtypes.

## 2. Materials and Methods

### 2.1. Study Participants

Pediatric participants were recruited from The Children’s Hospital, Zhejiang University School of Medicine. All samples were collected during the same respiratory infection season between November 2023 and January 2024. Inclusion criteria for MPP patients were as follows: (1) typical MPP symptoms (fever, cough, dyspnea, abnormal breath sounds); (2) radiographic evidence of pneumonia on chest imaging; (3) microbiological confirmation of MP infection by either positive MP RNA detection from nasopharyngeal (NP) swabs using nucleic acid amplification testing or a serum MP antibody titer ≥1:160; and (4) no antibiotic use within one month prior to sampling. Influenza A patients met similar criteria with positive influenza A (Flu) RNA detection. Healthy controls were individuals undergoing routine health examinations at the same hospital during the same study period. The controls had no respiratory or allergic symptoms within one month prior to sampling, no antibiotic use within one month, and no acute illness within one week after sampling. All NP swabs from controls were collected using the same standardized protocol used for patient groups. The participant recruitment, screening, exclusion, and inclusion process is summarized in Figure S1.

### 2.2. Sample Collection and Sequencing

Nasopharyngeal samples were collected using sterile swabs, which were rotated 3–5 times against the mucosa and stored in collection tubes (Hope Bio-Technology Co., Ltd., Qingdao, China) at −80 °C until DNA extraction. Microbial DNA was extracted using the E.Z.N.A.^®^ Soil Kit (Omega Bio-tek, GA, USA). For each extraction batch, a negative control containing nuclease-free water was processed alongside the samples through the entire DNA extraction procedure to monitor potential contamination. The V1-V9 region of the 16S rRNA gene was amplified using primers 27F (5′-AGRGTTYGATYMTGGCTCAG-3′) and 1492R (5′-RGYTACCTTGTTACGACTT-3′), with unique 8 bp barcode sequences assigned to each sample. PCR amplification was performed in triplicate 20 μL reactions containing 4 μL of 5× FastPfu Buffer, 2 μL of 2.5 mM dNTPs, 0.8 μL of each primer (5 μM), 0.4 μL of FastPfu Polymerase, and 10 ng of template DNA. Thermal cycling conditions were as follows: initial denaturation at 95 °C for 2 min; 27 cycles of 95 °C for 30 s, 55 °C for 30 s, and 72 °C for 60 s; and a final extension at 72 °C for 5 min. Amplicons were purified using the AxyPrep DNA Gel Extraction Kit (Axygen Biosciences, Union City, CA, USA). SMRTbell libraries were constructed according to the manufacturer’s instructions (Pacific Biosciences, Menlo Park, CA, USA) and sequenced on the PacBio Sequel II platform (Shanghai Biozeron Biotechnology Co. Ltd., Shanghai, China). PCR amplification included both no-template controls and extraction blank controls, and no substantial amplification products were detected in negative controls.

### 2.3. Bioinformatics Analysis

Raw PacBio reads were processed using SMRT Link Analysis v9.0 to generate circular consensus sequence (CCS) reads with the following parameters: minimum number of passes = 3 and minimum predicted accuracy = 0.99. Sequences shorter than 800 bp or longer than 2500 bp were removed. Additional filtering steps included the removal of barcode and primer sequences, chimeric sequences, and reads containing ≥10 consecutive identical bases. Sequencing depth distribution across samples and rarefaction curves are shown in Figure S2. Operational taxonomic units (OTUs) were clustered at 98.65% similarity using UPARSE v7.1, with chimeric sequences removed by UCHIME. Taxonomic classification was performed with RDP Classifier v2.2 against the Silva 16S rRNA database (SSU132) at a 70% confidence threshold. Alpha diversity (Shannon index) was analyzed using Mothur v1.21.1, and beta diversity (Bray–Curtis distance) was assessed via principal coordinate analysis (PCoA). Microbiota composition differences were evaluated using PERMANOVA (vegan package version 2.7.1 in R). Homogeneity of multivariate dispersion was assessed using betadisper analysis based on Bray–Curtis distances. Differences in microbiota composition among groups were further evaluated using multivariable regression models adjusted for age and sex. For alpha diversity, adjusted analyses were performed using multivariable linear regression models, controlling for age and sex. For beta diversity, adjusted PERMANOVA analyses were conducted based on Bray–Curtis distance matrices with age and sex included as covariates. LEfSe analysis was conducted via the Galaxy Huttenower Platform, and random forest (RF) models were constructed using the caret package in R (version 4.3.2) to classify MPP, Flu, and Normal based on nasopharyngeal microbiota profiles. To reduce potential confounding caused by age differences between the groups, microbial features were residualized with respect to age prior to RF classification. The resulting residualized microbial features were then used for model construction. To further assess whether classification performance depended primarily on *Mycoplasma* abundance, additional analyses were conducted after the exclusion of the *Mycoplasma* genus from the feature matrix. Model performance was evaluated using nested five-fold cross-validation, with inner cross-validation for parameter tuning and outer cross-validation for performance assessment. RF models were trained using 500 trees. Classification performance was assessed using confusion matrices, receiver operating characteristic (ROC) curves, and the area under the ROC curves (AUC) with 95% confidence intervals (CI). ROC analyses were performed in a one-vs-rest manner for each class (MPP, Flu, and Normal) using the pROC package. Additionally, sensitivity and specificity were calculated at a predefined probability threshold of 0.5. Variable importance was estimated using mean decrease accuracy (MDA) derived from RF models. Functional prediction analysis was performed using PICRUSt2 (v2.6.3) based on the KEGG database to infer the predicted functional potential of the microbiota in different groups. PICRUSt2 uses hidden-state prediction approaches based on reference genomes and reports nearest sequenced taxon index (NSTI) values as a quality metric reflecting the availability of closely related reference genomes for each sample. The median NSTI score was 0.06, which indicates that most bacteria within our samples had closely related reference genomes available for functional prediction. Differential pathway analyses were corrected for multiple testing using the Benjamini–Hochberg false discovery rate (FDR) method.

### 2.4. Statistical Analysis

All data were analyzed using R v4.0.2. Differences between two groups and three groups were tested using the Wilcoxon rank-sum test and Kruskal–Wallis rank-sum test, respectively. The accuracy of the diagnostic model was assessed using receiver operating characteristic (ROC) and area under the curve (AUC) analyses. All statistical tests were two-sided, and *p* < 0.05 was considered statistically significant.

## 3. Results

### 3.1. Characteristics of Study Participants

**Figure 1:**
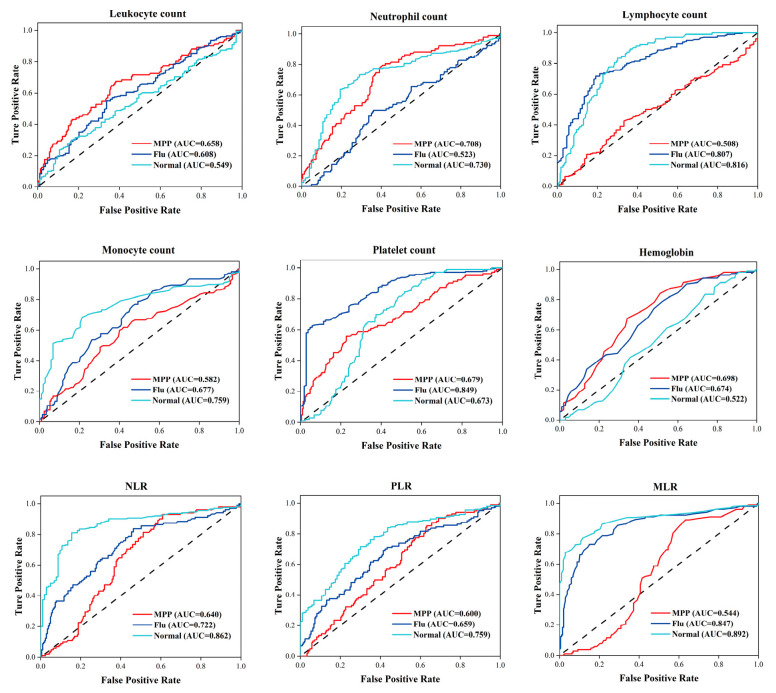
ROC curve analysis was performed to evaluate the classification performance of blood parameters in distinguishing MPP patients, Flu patients, and healthy controls.

The demographic and clinical characteristics of children with MPP and Flu and healthy controls are summarized in Table 1. The median age of MPP patients was 7.1 years, with a male-to-female ratio of 1:1. Significant differences in age were observed between the three groups (*p* < 0.001), whereas no significant difference in sex distribution was detected (*p* = 0.4). The biochemical analysis demonstrated significantly higher leukocyte and neutrophil counts in MPP patients compared with both Flu patients and healthy controls (*p* < 0.05, Table 1 and Table S1). C-reactive protein (CRP) was not measured in healthy controls; thus, only MPP and Flu groups were compared, with higher CRP levels observed in MPP patients (*p* < 0.05). Additionally, the neutrophil count, monocyte count, ratio of neutrophils to lymphocyte (NLR), ratio of platelet to lymphocyte (PLR), and ratio of monocyte to lymphocyte (MLR) were significantly elevated in both MPP and Flu patients relative to healthy controls (*p* < 0.05). The ROC curve analysis demonstrated that the neutrophil count and NLR exhibited the best discriminatory power, with AUC values greater than 0.64 for differentiating MPP, Flu, and healthy groups (Figure 1).

### 3.2. Alterations in Nasopharyngeal Microbiota of MPP Patients

**Figure 2:**
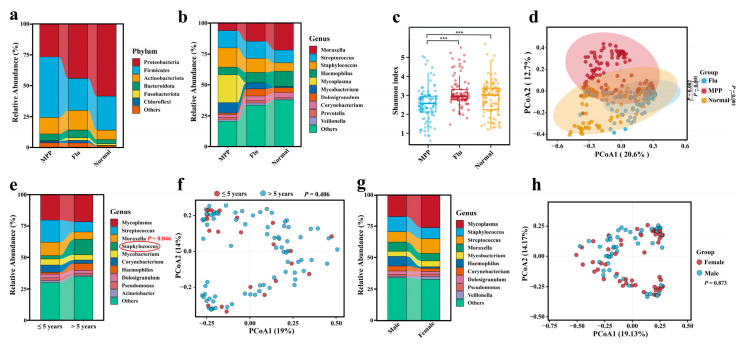
Comparison of the nasopharyngeal microbiota between MPP patients, Flu patients, and healthy controls. (**a**) Relative abundance of the microbiota at the phylum level and (**b**) at the genus level. (**c**) Alpha diversity assessed using the Shannon index. (**d**) Beta diversity evaluated through principal coordinate analysis based on Bray–Curtis distances. (**e**–**h**) Age- and gender-associated variations in the nasopharyngeal microbiota of MPP patients. (**e**) Genus-level relative abundance stratified by age groups. (**f**) Beta diversity comparison between age groups. (**g**) Genus-level relative abundance stratified by gender. (**h**) Beta diversity comparison between genders. *** *p* < 0.001.

To identify MPP-associated changes in the microbiota, we analyzed 16S rRNA sequencing data from 309 nasopharyngeal swab samples (102 MPP, 104 Flu, and 103 healthy controls). At the phylum level, Firmicutes, Proteobacteria, Actinobacteriota, and Bacteroidota dominated, accounting for 48.8%, 26.9%, 13.3%, and 5.4% of the MPP microbiota, respectively (Figure 2a). At the genus level, *Mycoplasma* (22.6%), *Staphylococcus* (15.8%), and *Streptococcus* (13.9%) were predominant in MPP patients, while *Moraxella* and *Streptococcus* were dominant in Flu patients and *Moraxella* and *Haemophilus* in healthy controls (Figure 2b). The alpha diversity (Shannon index) was significantly lower in MPP patients compared to Flu patients and healthy controls (*p* < 0.001, Figure 2c), indicating reduced alpha diversity. Beta diversity analysis (PCoA) revealed significant intergroup differences (*p* < 0.05, Figure 2d), confirming nasopharyngeal dysbiosis in MPP patients. No significant differences in multivariate dispersion were observed among groups (betadisper permutation test, *p* = 0.18), supporting the robustness of the PERMANOVA results. To evaluate the potential effects of age and sex on the microbiome results, multivariable adjustment analyses were performed for genus-level composition, alpha diversity, and beta diversity. The results revealed that the differential abundance patterns of the top 10 most abundant genera remained largely unchanged after adjustment (Table S1). In addition, the results of alpha diversity analyses were consistent before and after adjustments, with significant differences still observed between the MPP and Flu groups as well as between the MPP and Normal groups (both unadjusted and adjusted MPP_vs._Flu_*p* < 0.001; both unadjusted and adjusted MPP_vs_Normal_*p* < 0.001). Similarly, the PERMANOVA analysis based on Bray–Curtis dissimilarity demonstrated that beta diversity remained significantly different between groups before and after adjustment (both unadjusted and adjusted Pr(>F) = 0.001). In addition, variations in the nasopharyngeal microbiota were examined across different demographic subgroups of MPP patients, categorized by age (≤5 years and >5 years) and gender (male and female). The results indicated a significantly higher relative abundance of the genus *Staphylococcus* in younger children (≤5 years) with MPP (*p* = 0.046), whereas no significant gender-based differences were observed (Figure 2e,g). The analysis of β-diversity further confirmed that neither age nor gender exerted a significant influence on the microbial community structure (*p* > 0.05) (Figure 2f,h).

**Figure 3:**
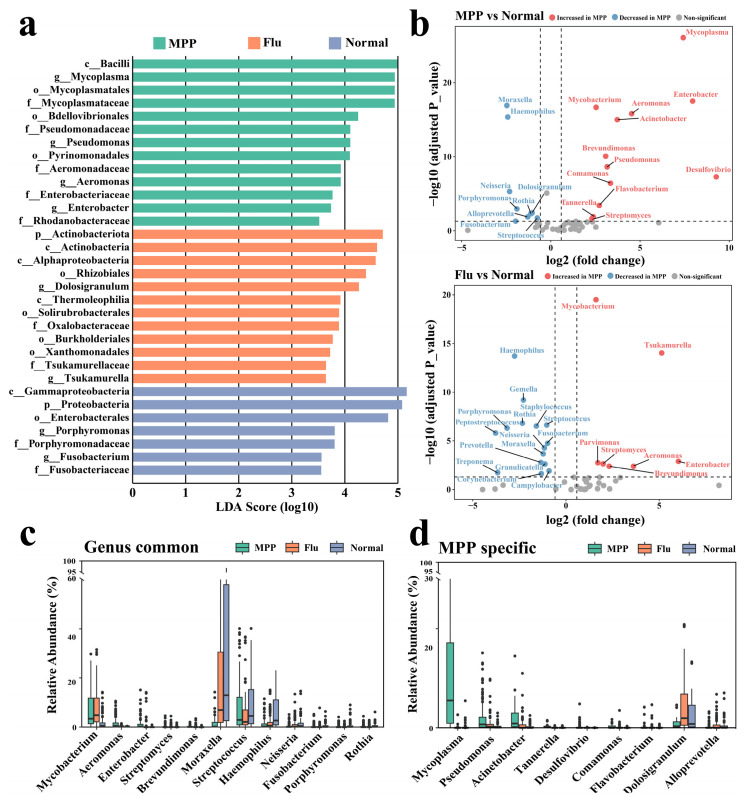
Microbial taxa with differential abundance in MPP patients, Flu patients, and healthy controls. (**a**) Taxa with significantly different abundances between MPP patients, Flu patients, and healthy controls identified by LEfSe analysis. (**b**) Volcano plots showing significantly different genera between MPP patients and healthy controls (top) and between Flu patients and healthy controls (bottom) (*p* < 0.05, 1.5× fold change cutoff). (**c**) Relative abundance of “common genera” and (**d**) “MPP specific” genera. The genera that are differentially expressed in both MPP and influenza patients compared to healthy controls are defined as “common genera”, while those differentially expressed only in MPP patients are defined as “MPP specific”.

The LEfSe analysis identified *Mycoplasma*, *Pseudomonas*, *Aeromonas*, and *Enterobacter* as enriched genera in MPP patients, while *Dolosigranulum* and *Tsukamurella* were enriched in Flu patients and *Porphyromonas* and *Fusobacterium* in healthy controls (Figure 3a). The differential abundance analysis highlighted 21 and 22 species with significant changes in MPP and Flu patients, respectively, compared to healthy controls (Figure 3b). Covariate-adjusted analyses controlling for age and sex yielded largely consistent results, with the majority of differentially abundant bacterial genera remaining significant after adjustment (Table S3). Shared features in both patient groups included higher abundances of *Mycobacterium*, *Aeromonas*, *Enterobacter*, *Streptomyces*, and *Brevundimonas* and lower abundances of *Moraxella*, *Streptococcus*, *Haemophilus*, *Neisseria*, *Fusobacterium*, *Porphyromonas*, and *Rothia* (Figure 3c). Unique to MPP patients were higher abundances of *Mycoplasma*, *Pseudomonas*, *Acinetobacter*, *Tannerella*, *Desulfovibrio*, *Comamonas*, and *Flavobacterium* and lower abundances of *Dolosigranulum* and *Alloprevotella* (Figure 3d). These findings reveal distinct nasopharyngeal microbial alterations in MPP patients, characterized by increased opportunistic pathogens like *Pseudomonas* and *Acinetobacter*, suggesting that MP infection is associated with changes in the microbial community composition.

### 3.3. Association Between the Nasopharyngeal Microbiota and MPP Subtypes

**Figure 4:**
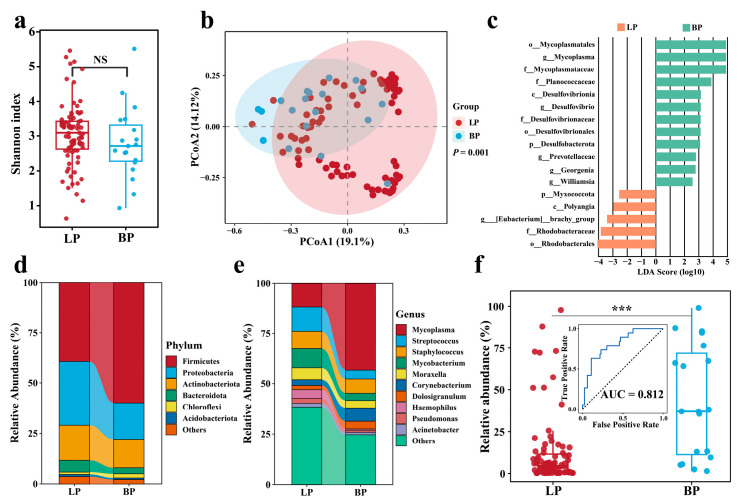
Comparison of nasopharyngeal microbiota between lobar pneumonia (LP) and bronchopneumonia (BP) in MPP patients. (**a**) Alpha diversity, (**b**) beta diversity, and (**c**) LEfSe analysis. (**d**) Relative abundance at the phylum level and (**e**) at the genus level. (**f**) Relative abundance of *Mycoplasma* and ROC curve analysis in LP and BP. *** *p* < 0.001.

MPP was classified into lobar pneumonia (LP, n = 83) and bronchopneumonia (BP, n = 19) to explore microbiota associations with MPP subtypes (Table S2). No significant difference in alpha diversity was observed between the LP and BP groups (*p* > 0.05, Figure 4a). Even after adjustments for age and sex using multivariable linear regression models, the alpha diversity was not significantly different between groups (adjusted *p* = 0.0649). In contrast, the beta diversity analysis revealed a significant difference in community composition (*p* = 0.001, Figure 4b), which remained significant after adjustments for age and sex (adjusted PERMANOVA, *p* = 0.001). No significant differences in multivariate dispersion were detected between groups (betadisper permutation test, *p* > 0.05), supporting the robustness of the beta diversity results. The LEfSe analysis revealed distinct microbial signatures between the two subtypes. BP patients exhibited significantly higher abundances of *Mycoplasma*, *Desulfovibrio*, *Prevotellaceae*, *Georgenia*, and *Williamsia*, while LP patients demonstrated a higher abundance of *[Eubacterium]_brachy_group* (Figure 4c). Furthermore, Firmicutes phyla were significantly more abundant in the BP group compared to LP (Figure 4d), and *Mycoplasma* was notably more abundant in BP (*p* < 0.001) (Figure 4e,f). The ROC analysis demonstrated that *Mycoplasma* could distinguish between LP and BP with an AUC of 0.812, suggesting its potential as a diagnostic biomarker for differentiating these two types of MPP (Figure 4f).

### 3.4. Identification of Microbial Classifiers for MPP

**Figure 5:**
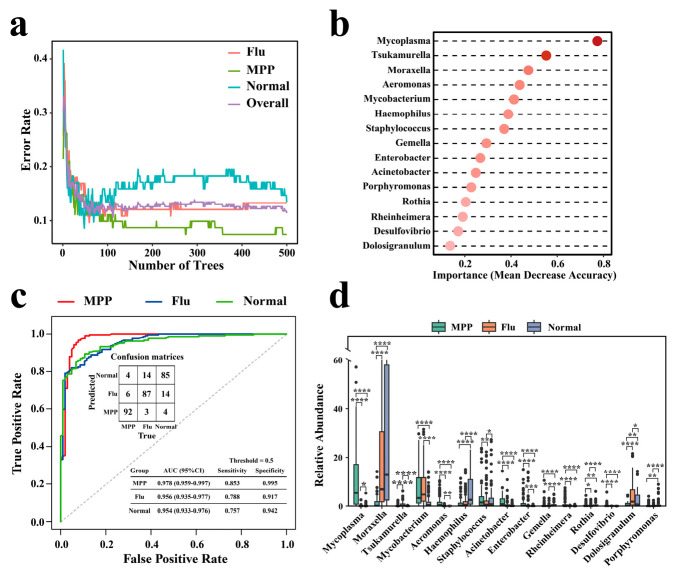
Key microbial genera for classifying MPP patients, Flu patients, and healthy controls identified by random forest analysis. (**a**) Cumulative error rate of random forest classification. (**b**) The 15 most significant microbial genera identified by random forest. (**c**) ROC curve analysis evaluating the classification performance for distinguishing MPP patients, Flu patients, and healthy controls. (**d**) Relative abundance of the top 15 key microbial genera. * *p* < 0.05, ** *p* < 0.01, *** *p* < 0.001, **** *p* < 0.0001.

To identify MPP-associated microbial biomarkers, a random forest model with 500 trees was constructed to identify MPP-associated microbial biomarkers. The out-of-bag (OOB) error was used as an internal estimate of model performance, yielding an overall error rate of 0.11 (Figure 5a). The random forest model identified the top 15 genera contributing most to classification, as determined by the mean decrease accuracy metric (Figure 5b). RF models demonstrated strong discriminatory performance among the MPP, Flu, and Normal groups (Figure 5c). In the primary model including all genera, the confusion matrix demonstrated a high classification accuracy, with 92/102 MPP, 87/104 Flu, and 85/103 Normal samples correctly classified. Misclassifications mainly occurred between Flu and Normal groups. The ROC analysis demonstrated an excellent classification performance, with AUC values of 0.978 (95% CI: 0.959–0.997) for MPP, 0.956 (95% CI: 0.935–0.977) for Flu, and 0.954 (95% CI: 0.933–0.976) for Normal. At the predefined probability threshold of 0.5, the RF model achieved sensitivities/specificities of 0.853/0.995 for MPP, 0.788/0.917 for Flu, and 0.757/0.942 for Normal, respectively. Compared to healthy controls, MPP patients exhibited higher relative abundances of *Mycoplasma*, *Mycobacterium*, *Aeromonas*, *Acinetobacter*, *Enterobacter*, *Gemella*, *Rheinheimera*, and *Desulfovibrio* and lower abundances of *Moraxella*, *Haemophilus*, *Rothia*, *Dolosigranulum*, and *Porphyromonas* (*p* < 0.05, KW test) (Figure 5d). These results reveal distinct microbial signatures associated with MPP, which may inform future studies aimed at developing diagnostic biomarkers. To further assess the potential impact of age imbalance, RF models were constructed using microbial features residualized with respect to age prior to classification (Figure S3a). The overall classification performance remained highly consistent with the primary analysis. The residualized RF model correctly classified 87/102 MPP, 86/104 Flu, and 85/103 Normal samples. Corresponding AUC values were 0.931 (95% CI: 0.891–0.970) for MPP, 0.936 (95% CI: 0.909–0.962) for Flu, and 0.928 (95% CI: 0.900–0.956) for Normal. Sensitivities/specificities at the 0.5 threshold were 0.843/0.966 for MPP, 0.779/0.922 for Flu, and 0.738/0.922 for Normal, respectively. These findings suggest that the classification performance was not solely driven by age-related microbial variation, although a residual influence of age cannot be completely excluded. To determine whether the classification performance was driven solely by the *Mycoplasma* abundance, RF analyses were repeated after excluding the *Mycoplasma* genus from the feature matrix (Figure S3b). Although the classification performance decreased moderately, the RF models retained a substantial discriminatory capacity. The model correctly classified 80/102 MPP, 87/104 Flu, and 85/103 Normal samples. AUC values remained high, reaching 0.949 (95% CI: 0.921–0.977) for MPP, 0.944 (95% CI: 0.918–0.969) for Flu, and 0.937 (95% CI: 0.911–0.964) for Normal. At the predefined probability threshold of 0.5, sensitivities/specificities were 0.775/0.986 for MPP, 0.798/0.917 for Flu, and 0.718/0.927 for Normal, respectively. These results suggested that non-*Mycoplasma* microbial dysbiosis also contributed substantially to disease classification. However, because an independent external validation cohort was not available in the current study, further multicenter validation studies are required to confirm the generalizability and clinical applicability of these models.

### 3.5. Predicted Functional Potential of the Nasopharyngeal Microbiota Associated with MPP

**Figure 6:**
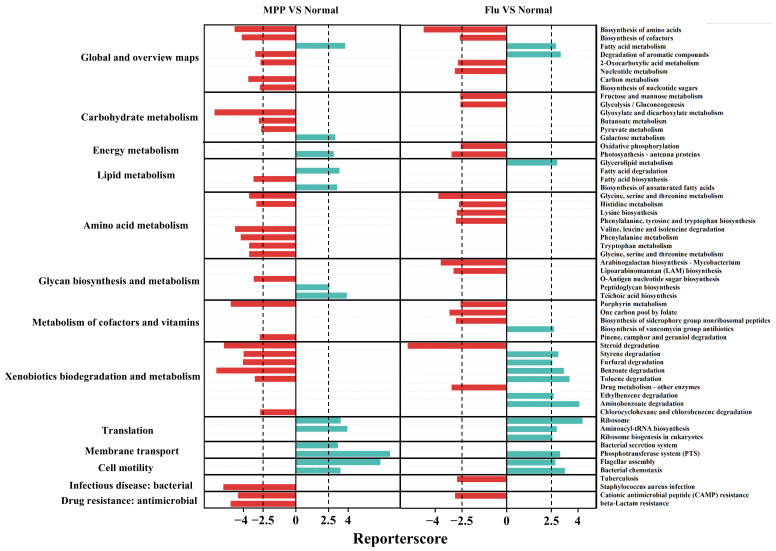
Differential enrichment of KEGG functions in MPP vs. Normal and Flu vs. Normal.

To further investigate functional changes in the nasopharyngeal microbiota associated with MPP, we predicted enriched KEGG pathways in the microbiota of MPP patients, Flu patients, and healthy controls (Figure 6). Nasopharyngeal samples from MPP patients exhibited higher metabolic potential for amino acids, including glycine, serine, threonine, histidine, valine, leucine, isoleucine, phenylalanine, and tryptophan. Among these, the metabolic pathways for glycine, serine, threonine, and histidine were uniquely enriched in MPP patients compared to Flu patients and healthy controls. These findings suggest that the nasopharyngeal microbiota of MPP patients prioritizes the metabolism of specific amino acids. Additionally, the microbiota of MPP patients displayed significant enrichment in pathways related to the biodegradation and metabolism of xenobiotic compounds, including the degradation of styrene, furfural, benzoate, and toluene, whereas pathways involved in drug metabolism were downregulated. Conversely, Flu patients exhibited higher energy metabolism potential (e.g., oxidative phosphorylation and photosynthesis-antenna proteins) compared to MPP patients and healthy controls. The microbiota of both MPP and Flu patients exhibited an enrichment in KEGG pathways associated with infectious disease; bacterial (e.g., tuberculosis and *Staphylococcus aureus* infection) and drug resistance; and antimicrobial resistance (e.g., cationic antimicrobial peptide (CAMP) resistance and beta-lactam resistance), suggesting altered predicted functional potentials associated with bacterial-infection-related and antimicrobial-resistance-related processes in the patient groups. In contrast, pathways related to lipid metabolism (e.g., glycerolipid metabolism, fatty acid biosynthesis, and biosynthesis of unsaturated fatty acids) and translation (e.g., ribosome, aminoacyl-tRNA biosynthesis, and ribosome biogenesis in eukaryotes) were relatively depleted in the nasopharyngeal microbiota of MPP and Flu patients. These findings suggest that the microbiota in these patient groups may have reduced capacities for lipid synthesis and genetic information processing. Finally, pathways related to membrane transport (e.g., bacterial secretion system and phosphotransferase system (PTS)) and cellular motility (e.g., flagellar assembly and bacterial chemotaxis) were significantly downregulated in the microbiota of both MPP and Flu patients. These data imply that the nasopharyngeal microbiota in MPP and Flu patients may exhibit diminished capabilities for the transport of ions, lipids, steroids, peptides, proteins, and carbohydrates across membranes, as well as impaired cellular motility.

## 4. Discussion

In this study, the nasopharyngeal microbiota of children with MPP were comprehensively characterized, and alterations associated with clinical subtypes and predicted functional profiles were identified. By leveraging high-throughput sequencing and advanced bioinformatic analysis, our findings provide valuable insights into the microbial dynamics associated with MPP and its subtypes. This study highlights the distinct microbial characteristics in children with MPP compared to healthy controls and discusses their potential associations with disease characteristics.

The nasopharyngeal microbiota in MPP patients demonstrated significant shifts in diversity and composition. Consistent with previous research, we observed a marked decrease in microbial diversity in the MPP group, which is characteristic of respiratory infections. The identification of specific microbial genera enriched in MPP patients, including *Mycoplasma*, *Pseudomonas*, *Acinetobacter*, *Tannerella*, *Desulfovibrio*, *Comamonas*, and *Flavobacterium*, highlights significant alterations in the nasopharyngeal microbiota associated with MPP. Among these, *Mycoplasma* is well-established as the primary pathogen in MPP, with its ability to evade the host immune system through variable surface proteins and its role in inducing inflammation by modulating cytokine production [2]. This genus is not only a hallmark of MPP but also a major taxonomic feature associated with the microbial alterations observed in the upper respiratory tract during infection [15]. *Pseudomonas* and *Acinetobacter* are both notable for their pathogenic potential in respiratory infections, often linked to hospital-acquired infections and chronic respiratory diseases [16]. In the context of MPP, the increased relative abundance of these taxa may be associated with alterations in the local microbial microenvironment and host immune responses, which could favor the persistence of specific microbial communities [17,18]. Notably, the presence of *Acinetobacter* has been reported in pediatric pneumonia cases with severe outcomes, suggesting a possible association with more severe disease manifestations [19]. Similarly, *Pseudomonas* is known for its production of virulence factors, such as exotoxins and proteases, which has previously been reported to produce virulence factors associated with epithelial disruption and inflammatory responses [20]. *Tannerella*, particularly *Tannerella forsythia*, is traditionally known as an oral anaerobic pathobiont but has increasingly been reported in lower respiratory tract infections, including pediatric and adult lung abscesses, suggesting that the disruption of the nasopharyngeal epithelial barrier during MPP may be associated with an increased relative abundance of this taxon and local inflammatory responses [21]. *Desulfovibrio*, a sulfate-reducing bacterium commonly associated with inflammation in the gastrointestinal tract, has recently been detected in extra-intestinal sites under disturbed host conditions. Its metabolites, such as hydrogen sulfide, have been reported to be associated with epithelial dysfunction and inflammatory responses, suggesting that the higher relative abundance of this taxon in the MPP microbiome may be associated with altered mucosal homeostasis [22,23]. *Comamonas*, an emerging opportunistic pathogen increasingly linked to respiratory, bloodstream, and hospital-acquired infections, possesses strong biofilm-forming and environmental persistence capabilities, and its appearance in MPP patients may be associated with alterations in the airway microbial microenvironment and host–microbiota interactions [24,25]. *Flavobacterium* (including species historically classified within the *Flavobacterium*–*Chryseobacterium* complex) is another opportunistic genus associated with pneumonia and nosocomial infections, particularly in children or individuals with compromised immunity. Its presence in the MPP group may reflect alterations in the microbial community composition associated with taxa previously reported to exhibit antimicrobial tolerance or inflammatory associations [26,27]. Collectively, the increased relative abundance of these genera suggests a broader microbial compositional shift in MPP beyond *Mycoplasma* dominance, which may be associated with altered host–microbiota interactions and disease severity. These findings suggest that the nasopharyngeal microbiota in MPP is characterized by complex compositional alterations, wherein *Mycoplasma* acts as the primary pathogen, while other enriched genera, such as *Pseudomonas*, *Acinetobacter*, *Tannerella*, *Desulfovibrio*, *Comamonas*, and *Flavobacterium*, may be associated with alterations in the microbial community structure and host–microbiota interactions. These observed changes align with previous research demonstrating that *Mycoplasma* infections are associated with alterations in the airway microbial community composition and shifts in the relative abundance of other taxa [10].

The clinical presentation and progression of MPP can be categorized into two major subtypes: lobar pneumonia (LP) and bronchopneumonia (BP). These subtypes exhibit distinct pathophysiological and microbiological characteristics. LP is characterized by confluent areas of focal alveolar disease, typically confined to a single lobe or segment of the lung. In contrast, BP demonstrates a multifocal distribution, with nodular lesions that often coalesce, leading to consolidation affecting one or more lung lobes [28]. The development of these subtypes is influenced by a range of factors, including the host immune response, the virulence of the pathogen, and environmental conditions [29]. In this study, distinct nasopharyngeal microbiota profiles were observed between LP and BP cases. Specifically, the relative abundance of *Mycoplasma* was significantly higher in BP compared to LP. This difference may be associated with variations in the disease presentation, host responses, and microbial community structure between the two pneumonia subtypes. BP is characterized by a more diffuse inflammation that spreads throughout the bronchial tree and affects multiple lobules of the lung. The widespread nature of BP may create a favorable environment for *M. pneumoniae*, which is known to thrive in areas of epithelial damage and impaired mucociliary clearance [30]. The diffuse inflammation in BP may weaken local immune defenses, which may be associated with the increased relative abundance of *Mycoplasma* compared to LP. Additionally, *Mycoplasma* possesses unique adhesins, such as P1 protein, which facilitate its attachment to the respiratory epithelium and evade host immune responses, promoting its persistence in BP cases [31]. In contrast, LP typically presents as a localized infection confined to one or more lung lobes, often associated with a robust and targeted immune response that limits bacterial proliferation. The lower abundance of *Mycoplasma* in LP may be due to the heightened immune activation in the affected lobes. Previous studies have shown that LP is frequently associated with infections by other bacterial pathogens, such as *Streptococcus pneumoniae* and *Klebsiella pneumoniae*, which may influence the microbial community composition and relative abundance patterns of *Mycoplasma* [32]. Differences in microbial community compositions may partially account for the reduced abundance of *Mycoplasma* in LP cases. Another explanation for the observed difference lies in the metabolic adaptability of *Mycoplasma*. *Mycoplasma* lacks a cell wall and relies on host-derived nutrients for survival. The microenvironment in BP, which involves widespread epithelial damage and increased nutrient leakage, may be associated with increased relative abundance of *Mycoplasma* than the relatively intact lung architecture observed in LP [33]. However, given the relatively small BP subgroup and the observational nature of this study, these findings should be interpreted cautiously and require validation in larger prospective studies.

In addition, the nasopharyngeal microbiota of MPP patients is characterized by significant alterations in amino acid metabolism and the degradation of other small molecules. It has been reported that amino acid imbalances can exacerbate epithelial immune responses, thereby increasing intestinal inflammation [34]. Similarly, the observed metabolic imbalances in the nasopharyngeal microbiota may contribute to changes in the immune microenvironment, potentially amplifying the burden of MPP. Our findings also revealed altered predicted functional profiles in MPP and Flu patients, including predicted pathways associated with bacterial infection (e.g., tuberculosis and *Staphylococcus aureus* infection) and antimicrobial-resistance-related processes (e.g., beta-lactam resistance and cationic antimicrobial peptide resistance). These data align with prior reports suggesting that changes in the respiratory microbiota during infection are associated with shifts in microbial community compositions and predicted antimicrobial-resistance-related functions [35]. The diminished capacity for membrane transport and cellular motility in the MPP and influenza microbiota, as evidenced by the depletion of bacterial secretion systems, phosphotransferase systems (PTSs), flagellar assembly, and bacterial chemotaxis, may further hinder microbial adaptability and nutrient acquisition in the host environment. These functional deficits have been previously associated with alterations in microbial community composition during respiratory infections [36]. In addition, because PICRUSt2 analyses infer predicted functional potential rather than directly measured microbial functions, these results should be interpreted cautiously and warrant further validation using shotgun metagenomics or targeted functional assays.

This study identified distinct alterations in the nasopharyngeal microbiota associated with MPP. By characterizing differentially abundant microbial taxa and predicted functional changes, our findings provide additional insight into microbiota profiles associated with MPP. These observations may also help inform future studies investigating the potential relationship between the respiratory microbiota and therapeutic strategies for MPP. Given the increasing prevalence of MPP and its complications, further exploration of these therapeutic strategies is warranted. Despite its strengths, our study has several limitations. First, the cross-sectional design precludes the establishment of causal relationships between microbial alterations and MPP. Longitudinal studies are required to track microbial changes throughout the disease course. Second, the reliance on 16S rRNA sequencing limits the resolution of the microbial identification and functional predictions. Metagenomic sequencing could provide more comprehensive insights into the microbial taxonomy and function. Finally, 16S rRNA gene analysis cannot distinguish between live and dead bacteria. After bacteria are killed, their DNA sequences can remain present in the nasopharynx, where they can be detected. It should be noted that this study is based on 16S rRNA gene sequencing data, which provide relative rather than absolute abundances. Therefore, changes in the *Mycoplasma* and other taxa reflect compositional shifts and do not necessarily indicate changes in absolute bacterial load or colonization status. Future studies using quantitative PCR or digital PCR methods are needed to validate these findings. This limitation may result in the detection of DNA from non-viable bacteria, potentially resulting in overestimation of microbial diversity and abundance. Future studies could address this issue by incorporating RNA-based sequencing or viability-specific methods to better characterize active microbial populations in MPP. In addition, this study employed OTU-based clustering for a full-length 16S rRNA sequence analysis rather than amplicon sequence variant (ASV)-based approaches. Although OTU-based methods remain widely used in full-length 16S rRNA microbiome studies, ASV-based approaches may provide an improved sequence resolution and error correction. Therefore, the use of an OTU-based analysis should be considered a methodological limitation of the present study.

## 5. Conclusions

This study reveals significant alterations in the nasopharyngeal microbiota of children with MPP, which are characterized by reduced microbial diversity and differences in microbial composition. These changes in the microbiota were associated with MPP and its clinical subtypes. Bronchopneumonia cases exhibited a higher relative abundance of *Mycoplasma* compared with lobar pneumonia. The distinct microbial signatures identified in different MPP subtypes may provide insights into disease heterogeneity and microbial community alterations associated with respiratory infections. Future large-scale prospective studies and mechanistic investigations are needed to further clarify the relationships between microbial alterations and disease progression in MPP. Overall, these findings provide additional insight into alterations in the nasopharyngeal microbiota during respiratory infection and may provide a basis for future research in this area.

## Figures and Tables

**Table 1:** Subject characteristics.

	MPP (*n* = 102)	Flu (*n* = 104)	Normal (*n* = 103)
Age, median (IQR)	7.1 (5.9–8.4)	9.35 (7.1–11.0)	5.4 (4.1–7.3)
≤5—No. (%)	19 (18.63%)	6 (5.77%)	44 (42.72%)
5–7.5—No. (%)	44 (43.14%)	25 (24.04%)	37 (35.92%)
7.5–10—No. (%)	28 (27.45%)	34 (32.69%)	16 (15.53%)
10–16—No. (%)	11 (10.78%)	39 (37.50%)	6 (5.83%)
Gender (male/female)	51/51	61/43	60/43
Blood result median (IQR)			
Leukocyte count (×10^9^/L, 4.00–12.00)	8.65 (6.74–11.27)	7.03 (5.45–8.59)	7.22 (5.92–8.81)
Neutrophil count (×10^9^/L, 1.50–7.80)	5.52 (4.39–7.87)	4.91 (3.18–6.06)	3.45 (2.76–4.27)
Lymphocyte count (×10^9^/L, 0.70–4.90)	2.00 (1.36–2.98)	1.31 (0.89–1.81)	3.04 (2.35–3.66)
Monocyte count (×10^9^/L, 0.10–1.50)	0.62 (0.42–0.77)	0.68 (0.51–0.82)	0.41 (0.34–0.51)
Platelet count (×10^9^/L, 100.00–400.00)	356.50 (270.00–416.25)	239.50 (203.00–280.75)	342.00 (288.00–385.00)
Hemoglobin (g/L, 110–155)	123.00 (117.00–130.00)	130.00 (126.00–137.75)	128.00 (123.00–133.00)
C-reactive protein (mg/L, 0–8.00)	11.40 (3.89–21.54)	3.91 (2.02–8.70)	-
NLR (ratio of neutrophils to lymphocyte)	2.91 (1.92–4.37)	3.54 (2.07–6.69)	1.15 (0.87–1.53)
PLR (ratio of platelet to lymphocyte)	158.82 (130.00–219.06)	174.13 (132.83–272.74)	110.67 (88.86–147.26)
MLR (ratio of monocyte to lymphocyte)	0.31 (0.19–0.39)	0.53 (0.36–0.72)	0.14 (0.11–0.18)

## Data Availability

Sequencing data in this study are available in the National Center for Biotechnology Information (NCBI) Sequence Read Archive (SRA) under accession number PRJNA1295150.

## Supplementary Materials

The following supporting information can be downloaded at https://www.mdpi.com/article/10.3390/microorganisms14061374/s1: Table S1: Multivariable-adjusted microbiome analyses controlling for age and sex, Table S2: Clinical characteristics of MPP patients, Flu patients and healthy population, Table S3: Comparison of unadjusted and age/sex-adjusted differential abundance analyses of bacterial genera between groups, Figure S1: Flow diagram of participant screening, enrollment, and inclusion in the study, Figure S2: Sequencing depth distribution (A) and rarefaction curves (B) of samples, and Figure S3: Performance of age-adjusted (a) and *Mycoplasma*-excluded (b) random forest classification models.

## Abbreviations

The following abbreviations are used in this manuscript:

MPP	*Mycoplasma pneumoniae* pneumonia
BP	Bronchopneumonia
LP	Lobar pneumonia
MP	*Mycoplasma pneumoniae*
ARIs	Acute respiratory infections
LRTI	Lower respiratory tract infection
NP	Nasopharyngeal
OP	Oropharyngeal
rRNA	Ribosomal RNA
Flu	Influenza A
OTUs	Operational taxonomic units
PCoA	Principal coordinate analysis
ROC	Receiver operating characteristic
AUC	Area under the curve
NLR	Ratio of neutrophils to lymphocyte
PLR	Ratio of platelet to lymphocyte
MLR	Ratio of monocyte to lymphocyte
CAMP	Cationic antimicrobial peptide
PTS	Phosphotransferase system
