# Supplementary material for: Characterization of Nasopharyngeal Microbiota Dysbiosis in Children with Mycoplasma pneumoniae Pneumonia
Source: Microorganisms. 2026 Jun 21;14(6):1374. doi: 10.3390/microorganisms14061374 (PMC13305937; doi:10.3390/microorganisms14061374)
Supplement: Supplementary file 1 [file microorganisms-14-01374-s001.zip › microorganisms-4304778-supplementary.pdf]

Table S1: Multivariable-adjusted microbiome analyses controlling for age and sex

Genus	Unadjusted FDR	Adjusted FDR
Moraxella	2.14E-08	7.66E-10
Streptococcus	0.136	0.483
Staphylococcus	1.35E-05	4.83E-04
Haemophilus	5.49E-09	2.63E-08
Mycoplasma	2.32E-16	2.87E-14
Mycobacterium	2.48E-18	6.22E-17
Dolosigranulum	2.83E-05	9.20E-03
Corynebacterium	0.457	0.483
Prevotella	0.138	0.581
Veillonella	0.914	0.483

Table S2: Clinical characteristics of MPP patients, Flu patients and healthy population

ID	Gender	Age	Type of disease	MPP type	MPP diagnostic criteria (PCR/serology)	Leukocyte count (×10^9/L, 4.00–12.00)	Neutrophil count (×10^9/L, 1.50–7.80)	Lymphocyte count (×10^9/L, 0.70–4.90)	Monocyte count (×10^9/L, 0.10–1.50)	Platelet count (×10^9/L, 100–400)	Hemoglobin (g/L, 110–155)	C-reactive protein (mg/L, 0–8.00)	NLR	PLR	MLR
B001	female	6.1	MPP	LP	Both	11.33	6.11	4.44	0.74	294	118	1.29	1.38	66.22	0.17
B002	male	5	MPP	LP	PCR	7.99	4.35	3.05	0.54	248	117	5.49	1.43	81.31	0.18
B003	female	5.5	MPP	LP	Both	11.74	9.18	1.95	0.59	422	111	2.63	4.71	216.41	0.3
B004	male	6.3	MPP	LP	PCR	12.12	7.2	3.66	1.01	485	114	0.6	1.97	132.51	0.28
B005	male	12.9	MPP	LP	Both	7.38	4.77	1.31	1.27	379	145	16.92	3.64	289.31	0.97
B006	female	10	MPP	LP	Both	6.29	4.49	1.39	0.33	177	123	77.52	3.23	127.34	0.24
B007	female	4.1	MPP	LP	Both	18.31	0.33	6.71	1.25	448	109	3.73	0.05	66.77	0.19
B008	male	6.4	MPP	LP	Both	13.81	9.5	2.76	1.22	371	128	16.58	3.44	134.42	0.44
B009	male	5	MPP	BP	Both	12.52	8.42	2.24	0.86	388	121	26.42	3.76	173.21	0.38
B010	female	11.6	MPP	BP	Both	11.47	8.9	1.93	0.6	402	128	32.88	4.61	208.29	0.31
B011	male	4	MPP	BP	Both	9.53	5.15	2.92	1.03	382	112	6.95	1.76	130.82	0.35
B012	female	6.9	MPP	BP	Both	7	5.65	1.23	0.11	268	139	13.02	4.59	217.89	0.09
B013	female	6	MPP	LP	PCR	4.17	2.18	1.58	0.4	242	139	14.99	1.38	153.16	0.25
B014	male	3.2	MPP	BP	Both	5.32	4.08	1.11	0.12	276	133	11.32	3.68	248.65	0.11
B015	female	6.9	MPP	LP	PCR	5.46	3.05	2.02	0.37	237	117	19.05	1.51	117.33	0.18
B016	male	10.2	MPP	BP	Both	7.33	4.81	1.7	0.81	159	109	28.53	2.83	93.53	0.48
B017	male	9.8	MPP	LP	Both	11.25	8.29	1.89	1.03	389	118	32.88	4.39	205.82	0.54
B018	male	3.9	MPP	LP	Serology	10.39	5.81	3.1	0.95	315	116	8.23	1.87	101.61	0.31
B019	male	6.1	MPP	LP	Both	7.96	5.56	1.75	0.64	383	122	13.51	3.18	218.86	0.37
B020	male	8.4	MPP	LP	Both	7.76	5.48	1.49	0.59	225	121	21.47	3.68	151.01	0.4
B021	male	7	MPP	LP	PCR	5.68	4.21	0.92	0.53	390	113	13.86	4.58	423.91	0.58
B022	female	8.1	MPP	BP	Both	10.18	8.12	1.81	0.22	430	125	1.91	4.49	237.57	0.12
B023	male	6.1	MPP	BP	Both	11.7	6.66	3.94	0.76	541	134	5.5	1.69	137.31	0.19
B024	male	7.1	MPP	LP	PCR	10.39	5.55	3.86	0.74	457	119	0.7	1.44	118.39	0.19

B025	male	7.1	MPP	LP	Both	9.83	7.66	1.35	0.75	429	119	23.62	5.67	317.78	0.56
B026	male	8	MPP	BP	Both	9.8	7.78	1.68	0.3	395	105	20.23	4.63	235.12	0.18
B027	male	7.3	MPP	BP	Both	8.71	5.89	1.44	1.35	259	117	0.23	4.09	179.86	0.94
B028	female	7	MPP	LP	Both	10.94	7.63	2.28	0.84	488	117	19.31	3.35	214.04	0.37
B029	female	7.8	MPP	BP	Both	7.83	6.51	0.91	0.23	271	100	22.75	7.15	297.8	0.25
B030	male	8.5	MPP	BP	Both	10.04	6.41	2.54	0.69	391	129	8.27	2.52	153.94	0.27
B031	female	8.6	MPP	LP	Both	4.82	3.16	1.17	0.24	294	126	3.94	2.7	251.28	0.21
B032	female	6.4	MPP	LP	Both	4.97	2.65	1.88	0.44	264	119	7.36	1.41	140.43	0.23
B033	male	11.2	MPP	LP	Both	6.69	4.97	1.14	0.54	275	126	18.82	4.36	241.23	0.47
B034	female	9.9	MPP	LP	Both	15.9	10	5.01	0.75	668	129	9.71	2	133.33	0.15
B035	male	7	MPP	LP	Both	4.74	2.15	2.06	0.25	297	130	4.69	1.04	144.17	0.12
B036	female	3.4	MPP	LP	Serology	14.98	12.66	1.54	0.76	224	108	107.42	8.22	145.45	0.49
B037	male	9.8	MPP	LP	Both	8.31	6.39	1.32	0.58	253	122	77.41	4.84	191.67	0.44
B038	male	7.7	MPP	LP	Both	11.1	6.79	3.51	0.58	493	140	2.11	1.93	140.46	0.17
B039	male	5.5	MPP	BP	Both	10.54	6.07	3.26	0.74	353	121	3.99	1.86	108.28	0.23
B040	female	7	MPP	LP	Both	6.79	3.97	2.02	0.75	349	124	56.11	1.97	172.77	0.37
B041	male	6.9	MPP	LP	Both	9.27	4.27	4.13	0.36	486	110	2.75	1.03	117.68	0.09
B042	female	9.6	MPP	LP	Both	11.13	8.48	2.36	0.26	357	143	5.23	3.59	151.27	0.11
B043	female	10.1	MPP	LP	Both	5.14	3.85	1.09	0.19	283	120	3.64	3.53	259.63	0.17
B044	male	8.1	MPP	LP	Both	8.46	4.71	3.14	0.58	363	121	1.68	1.5	115.61	0.18
B045	male	4.4	MPP	LP	Serology	13	9.21	3.47	0.29	411	138	0.2	2.65	118.44	0.08
B046	female	7.5	MPP	LP	Serology	9.47	8.4	0.83	0.23	359	133	6.59	10.12	432.53	0.28
B047	female	7	MPP	LP	Both	13.31	10.12	2.33	0.76	446	115	2.34	4.34	191.42	0.33
B048	male	10.3	MPP	LP	Serology	6.51	5.22	0.56	0.71	242	130	6.98	9.32	432.14	1.27
B049	male	4.2	MPP	LP	Both	9.61	4.69	4.1	0.69	413	125	27.49	1.14	100.73	0.17
B050	male	4	MPP	LP	Both	8.06	5.47	2.03	0.38	288	125	11.62	2.69	141.87	0.19
B051	male	7.5	MPP	LP	Both	8.17	5.05	2.39	0.7	335	133	3.04	2.11	140.17	0.29
B052	male	2.7	MPP	BP	Both	4.34	1.79	2.11	0.43	298	113	13.46	0.85	141.23	0.2
B053	male	4	MPP	LP	Both	61.37	56.58	2.95	1.84	347	124	89.77	19.18	117.63	0.62
B054	female	8.9	MPP	LP	Both	8.78	4.4	36.1	0.72	459	139	4.9	0.12	12.71	0.02
B055	female	7.5	MPP	BP	PCR	15.45	10.81	3.63	0.85	480	119	7.29	2.98	132.23	0.23
B056	male	7	MPP	LP	Both	5.8	4.3	1.17	0.28	257	130	13.94	3.68	219.66	0.24

B057	male	7.1	MPP	LP	Both	15.9	11.85	3.31	0.64	500	140	0.3	3.58	151.06	0.19
B058	male	9.3	MPP	LP	Both	9.01	5.76	2.3	0.73	289	109	10.13	2.5	125.65	0.32
B059	female	8.7	MPP	LP	Both	7.74	4.86	1.77	0.6	265	114	10.25	2.75	149.72	0.34
B060	male	7.2	MPP	LP	Both	6.67	4.34	1.58	0.57	256	125	10.64	2.75	162.03	0.36
B061	female	6.9	MPP	LP	PCR	16.3	10.98	3.44	1.86	390	137	39.8	3.19	113.37	0.54
B062	female	5.1	MPP	LP	Both	8.09	4.71	1.98	0.78	343	126	21.75	2.38	173.23	0.39
B063	female	8	MPP	LP	Both	6.06	4.95	0.78	0.31	319	131	15.37	6.35	408.97	0.4
B064	male	6.7	MPP	LP	Serology	6.35	3.97	1.39	0.78	463	126	18.46	2.86	333.09	0.56
B065	male	11.2	MPP	LP	Both	10.22	4.55	2.69	1.14	554	118	14.55	1.69	205.95	0.42
B066	male	4.8	MPP	LP	Both	12.86	6.8	4.41	1.61	617	134	0.58	1.54	139.91	0.37
B067	female	8.9	MPP	LP	Both	7.62	5.89	1.23	0.46	247	106	16.29	4.79	200.81	0.37
B068	male	11	MPP	LP	Both	6.25	4.65	0.99	0.43	162	138	30.82	4.7	163.64	0.43
B069	male	4.1	MPP	LP	Both	9.33	6.39	2.4	0.54	415	132	9.04	2.66	172.92	0.23
B070	female	8.3	MPP	BP	Both	10.25	7.75	2.14	0.69	348	124	23.54	3.62	162.62	0.32
B071	female	8	MPP	LP	PCR	11.76	9.67	1.26	0.83	270	119	69.94	7.67	214.29	0.66
B072	female	9.5	MPP	LP	Both	11.36	7.01	3.31	0.92	356	140	3.16	2.12	107.55	0.28
B073	female	12	MPP	BP	Both	9.8	4.65	2.21	0.68	312	126	24.65	2.1	141.18	0.31
B074	female	7.1	MPP	LP	Both	3.98	2.86	0.71	0.41	246	123	74.21	4.03	346.48	0.58
B075	male	4.1	MPP	LP	Both	6.11	3.74	1.48	0.49	348	127	21.14	2.53	235.14	0.33
B076	female	5.1	MPP	LP	Both	8.15	4.89	2.54	0.59	500	132	1.31	1.93	196.85	0.23
B077	female	6.3	MPP	LP	PCR	19.89	17.76	1.41	0.72	501	132	26.09	12.6	355.32	0.51
B078	male	4.5	MPP	LP	Both	8.01	3.21	4.01	0.77	336	129	1.33	0.8	83.79	0.19
B079	female	6.9	MPP	LP	Both	9.54	5.11	3.42	0.84	398	111	17.8	1.49	116.37	0.25
B080	female	6.2	MPP	LP	Both	10.71	6.91	2.06	0.96	409	109	37.54	3.35	198.54	0.47
B081	female	7.6	MPP	LP	PCR	7.39	3.65	2.68	0.54	417	122	0.66	1.36	155.6	0.2
B082	male	5.4	MPP	LP	Both	8.22	4.51	2.87	0.58	475	117	36.74	1.57	165.51	0.2
B083	female	9.6	MPP	LP	Both	4.58	3.05	1.19	0.33	135	121	14.55	2.56	113.45	0.28
B084	female	3.3	MPP	LP	Both	8.42	6.4	1.67	0.35	213	110	223.56	3.83	127.54	0.21
B085	female	4.2	MPP	LP	Both	8.32	5.2	2.42	0.67	424	113	0.37	2.15	175.21	0.28
B086	female	7.1	MPP	LP	Both	5.65	3.86	1.18	0.39	442	122	2.6	3.27	374.58	0.33
B087	female	6	MPP	LP	Serology	15.89	8.9	5.86	1.08	357	126	0.64	1.52	60.92	0.18
B088	female	6.1	MPP	BP	PCR	4.19	2.29	1.36	0.46	193	117	19.53	1.68	141.91	0.34

B089	female	8.3	MPP	LP	Both	8.75	5.73	1.97	0.66	291	137	5.2	2.91	147.72	0.34
B090	female	5.7	MPP	LP	Both	5.63	3.57	1.47	0.56	527	116	0.84	2.43	358.5	0.38
B091	female	7.2	MPP	LP	Both	22.03	17.69	3.26	1.01	360	130	11.48	5.43	110.43	0.31
B092	male	4.1	MPP	LP	Both	6.1	4.72	0.95	0.39	372	112	10.27	4.97	391.58	0.41
B093	female	7.4	MPP	LP	Both	14.77	9.88	4.25	0.59	416	122	3.58	2.32	97.88	0.14
B094	male	8.1	MPP	LP	Both	12.35	8.26	2.84	1	382	121	33.65	2.91	134.51	0.35
B095	male	7.5	MPP	LP	Both	3.66	2.41	0.82	0.42	259	128	72.21	2.94	315.85	0.51
B096	female	14.7	MPP	BP	Both	6.75	5.02	1.25	0.45	229	136	8.8	4.02	183.2	0.36
B097	male	6.6	MPP	LP	Both	8.02	5.9	1.68	0.41	240	117	14.76	3.51	142.86	0.24
B098	female	13.2	MPP	LP	Both	8.02	4.91	2.19	0.67	373	134	8.34	2.24	170.32	0.31
B099	male	8.4	MPP	LP	Both	14.41	12.74	1.46	0.16	386	124	14.12	8.73	264.38	0.11
B100	female	9.7	MPP	LP	Both	8.64	8.54	1.14	0.45	308	119	10.56	7.49	270.18	0.39
B101	male	7.9	MPP	LP	Both	6.85	5.45	0.75	0.63	288	123	11.27	7.27	384	0.84
B102	female	7.3	MPP	LP	Both	8.65	8.77	1.66	0.65	270	128	15.98	5.28	162.65	0.39
C001	male	13.1	Flu			10.7	8.14	1.21	0.86	296	141	11.68	6.73	244.63	0.71
C002	female	13	Flu			7.28	4.2	2.29	0.77	131	141	4.01	1.83	57.21	0.34
C003	female	12	Flu			7.5	5.49	1.26	0.69	262	130	5.49	4.36	207.94	0.55
C004	male	13.9	Flu			5.19	2.37	2.12	0.66	187	163	15.62	1.12	88.21	0.31
C005	female	13.1	Flu			10.6	7.94	1.86	0.73	214	140	10.74	4.27	115.05	0.39
C006	male	11.2	Flu			6.93	4.26	1.82	0.78	211	126	16.7	2.34	115.93	0.43
C007	female	10	Flu			7.55	6.64	0.48	0.41	206	128	9.99	13.83	429.17	0.85
C008	female	13.1	Flu			7.69	5.44	1.73	0.38	399	106	1.22	3.14	230.64	0.22
C009	male	13.8	Flu			4.65	3.17	1	0.04	175	162	18.69	3.17	175	0.04
C010	male	7	Flu			3.62	1.71	1.42	0.43	243	137	4.52	1.2	171.13	0.3
C011	female	11.7	Flu			6.54	4.54	1.37	0.59	194	126	1.26	3.31	141.61	0.43
C012	male	10.1	Flu			6.65	4.11	1.72	0.74	270	145	0.57	2.39	156.98	0.43
C013	female	13.2	Flu			7.54	5.12	1.78	0.76	266	141	3.66	2.88	149.44	0.43
C014	female	10.5	Flu			8.8	6.17	1.8	0.68	264	147	11.92	3.43	146.67	0.38
C015	male	9.7	Flu			7.73	5.97	1.12	0.59	189	131	2.17	5.33	168.75	0.53
C016	male	11.7	Flu			9.44	7.5	0.99	0.76	330	130	14.29	7.58	333.33	0.77
C017	male	7	Flu			11.1	8.64	1.28	1.15	192	169	17.94	6.75	150	0.9
C018	male	10.5	Flu			7.6	5.16	1.75	0.63	280	127	3.11	2.95	160	0.36

C019	male	9.3	Flu			7.71	3.42	3.14	0.83	280	129	4.87	1.09	89.17	0.26
C020	male	7.9	Flu			9.52	7.98	0.89	0.62	244	134	15.37	8.97	274.16	0.7
C021	male	10.2	Flu			5.41	4.3	0.52	0.5	161	118	3.95	8.27	309.62	0.96
C022	female	10.8	Flu			9.36	5.76	2.42	1.15	228	138	4.17	2.38	94.21	0.48
C023	female	9.7	Flu			4.37	3.02	0.86	0.48	149	134	2.09	3.51	173.26	0.56
C024	male	9.6	Flu			6.85	5.61	0.55	0.67	261	139	7.7	10.2	474.55	1.22
C025	male	9.6	Flu			5.41	3.78	0.89	0.52	260	130	1.89	4.25	292.13	0.58
C026	male	11.3	Flu			8.28	5.71	1.6	0.8	292	129	3.51	3.57	182.5	0.5
C027	male	12.2	Flu			10.37	6.08	2.96	0.85	290	132	0.55	2.05	97.97	0.29
C028	male	8.9	Flu			8.08	5.35	1.91	0.77	205	141	4.29	2.8	107.33	0.4
C029	male	9	Flu			3.64	2.6	0.79	0.24	181	128	0.03	3.29	229.11	0.3
C030	female	10.5	Flu			5.74	1.4	3.82	0.42	240	137	1.26	0.37	62.83	0.11
C031	male	11.4	Flu			11.24	8.71	1.25	0.88	239	135	10.76	6.97	191.2	0.7
C032	female	9.6	Flu			7.05	5.23	1.16	0.59	203	131	5.45	4.51	175	0.51
C033	male	9.3	Flu			5.58	3.22	1.51	0.61	300	124	1.85	2.13	198.68	0.4
C034	female	11.7	Flu			5.62	4.05	1.01	0.54	278	141	2.97	4.01	275.25	0.53
C035	male	15.1	Flu			6.35	4.46	1.35	0.52	195	154	11.21	3.3	144.44	0.39
C036	male	8.1	Flu			13.43	9.04	3.49	0.68	290	129	2.69	2.59	83.09	0.19
C037	male	8	Flu			4.54	2.46	1.29	0.67	188	121	7.31	1.91	145.74	0.52
C038	female	9	Flu			8.38	6.54	0.91	0.85	265	129	3.56	7.19	291.21	0.93
C039	male	8	Flu			30	0.51	3.66	0.11	8.1	39.6	5.69	0.14	2.21	0.03
C040	female	9	Flu			8.01	5.88	1.3	0.81	192	133	6.94	4.52	147.69	0.62
C041	female	8	Flu			10.33	6.17	2.8	0.69	446	114	18.39	2.2	159.29	0.25
C042	male	11	Flu			6.26	2.97	2.81	0.37	456	127	0.2	1.06	162.28	0.13
C043	female	9.1	Flu			8.63	5.96	1.58	0.95	289	128	2.52	3.77	182.91	0.6
C044	male	7	Flu			6.55	5.18	0.79	0.48	245	131	0.53	6.56	310.13	0.61
C045	male	10.2	Flu			8.75	6.75	0.86	1.13	201	152	8.28	7.85	233.72	1.31
C046	male	7.5	Flu			9.71	7.04	1.24	1.38	298	126	0.92	5.68	240.32	1.11
C047	female	10	Flu			5.28	3.02	1.67	0.56	256	139	2.15	1.81	153.29	0.34
C048	female	7.1	Flu			10.47	8.82	0.81	0.79	312	141	0.56	10.89	385.19	0.98
C049	female	9.4	Flu			6.04	3.23	1.63	0.95	294	133	3.86	1.98	180.37	0.58
C050	female	13.1	Flu			7.19	5.62	0.74	0.81	152	137	3	7.59	205.41	1.09

C051	female	7.1	Flu			4.8	2.84	1.48	0.44	206	136	1.09	1.92	139.19	0.3
C052	male	7.6	Flu			4.29	2.52	1.03	0.71	169	124	11.34	2.45	164.08	0.69
C053	female	7.1	Flu			10.84	9.17	0.91	0.73	304	123	19.46	10.08	334.07	0.8
C054	male	7.2	Flu			10.59	7.83	1.78	0.82	227	134	1.79	4.4	127.53	0.46
C055	male	9.8	Flu			4.86	2.78	1.32	0.72	242	131	1.85	2.11	183.33	0.55
C056	male	9.4	Flu			4.98	2.98	1.56	0.77	231	134	2.69	1.91	148.08	0.49
C057	female	6.2	Flu			5.25	3.12	1.21	0.69	239	129	1.99	2.58	197.52	0.57
C058	female	6.5	Flu			5.91	3.36	1.7	0.61	224	135	2.38	1.98	131.76	0.36
C059	female	6.5	Flu			8.27	6.98	0.89	0.36	289	126	6.71	7.84	324.72	0.4
C060	male	6.5	Flu			6.91	4.08	1.9	0.82	318	125	12.33	2.15	167.37	0.43
C061	female	10.2	Flu			5.18	4.23	0.57	0.37	281	129	6.17	7.42	492.98	0.65
C062	female	6.6	Flu			6.68	5.25	0.69	0.55	277	131	7.71	7.61	401.45	0.8
C063	male	7.1	Flu			8.82	6.96	0.97	0.84	254	129	8.84	7.18	261.86	0.87
C064	male	7	Flu			8.4	9.45	2.49	0.86	226	136	1.13	3.8	90.76	0.35
C065	female	6.6	Flu			8.4	5.64	1.86	0.86	209	130	1.86	3.03	112.37	0.46
C066	male	6.3	Flu			11.01	9.53	0.57	0.73	312	129	10.04	16.72	547.37	1.28
C067	female	7.8	Flu			8.38	6.87	0.79	0.7	224	121	11.36	8.7	283.54	0.89
C068	male	5.4	Flu			6.37	4.18	1.42	0.51	310	127	2.37	2.94	218.31	0.36
C069	male	6.7	Flu			9.3	3.35	3.94	1.83	270	128	10.06	0.85	68.53	0.46
C070	female	7	Flu			7.18	5.87	0.74	0.55	312	133	1.74	7.93	421.62	0.74
C071	female	5	Flu			6.01	4.7	0.74	0.54	194	124	2.66	6.35	262.16	0.73
C072	male	10.9	Flu			6.98	5.24	1.16	0.54	295	128	2.2	4.52	254.31	0.47
C073	male	8.1	Flu			2.82	1.07	1.48	0.21	115	123	2.26	0.72	77.7	0.14
C074	male	6.1	Flu			7	5.53	0.89	0.51	311	116	2.21	6.21	349.44	0.57
C075	female	9.3	Flu			4.03	1.57	1.72	0.42	206	126	0.37	0.91	119.77	0.24
C076	male	7.1	Flu			4.47	2.2	1.48	0.78	237	140	6.66	1.49	160.14	0.53
C077	female	4.8	Flu			7.3	5.58	1.15	0.55	275	130	2.78	4.85	239.13	0.48
C078	female	13.1	Flu			11.77	8.07	2.24	1.39	297	135	25.58	3.6	132.59	0.62
C079	male	10.9	Flu			4.86	3.73	0.73	0.28	196	128	5.98	5.11	268.49	0.38
C080	male	12	Flu			7.43	4.05	2.9	0.44	238	121	1.56	1.4	82.07	0.15
C081	male	4.1	Flu			6.27	2.22	3.5	0.46	135	127	0.93	0.63	38.57	0.13
C082	male	4.7	Flu			10.99	9.24	0.77	0.97	227	125	2.47	12	294.81	1.26

C083	male	9.3	Flu			6.93	4.05	1.35	0.84	211	135	0.29	3	156.3	0.62
C084	male	11.3	Flu			4.28	1.07	2.82	0.29	234	140	1.12	0.38	82.98	0.1
C085	female	7	Flu			4.39	3.87	0.22	0.27	207	133	1.16	17.59	940.91	1.23
C086	male	3	Flu			6.09	3.37	2.03	0.57	321	123	1.95	1.66	158.13	0.28
C087	male	8.1	Flu			5.4	2.98	1.52	0.88	203	127	2.33	1.96	133.55	0.58
C088	male	9	Flu			12.23	10.66	0.66	0.84	300	123	3.6	16.15	454.55	1.27
C089	female	10	Flu			8.97	5.85	1.95	1.1	248	129	9.77	3	127.18	0.56
C090	male	10.9	Flu			11.42	8.66	1.58	1.14	191	138	3.73	5.48	120.89	0.72
C091	female	16	Flu			5.18	4.06	0.55	0.54	181	123	4.85	7.38	329.09	0.98
C092	male	13.5	Flu			6.98	4.55	1.25	0.82	249	133	6.56	3.64	199.2	0.66
C093	male	10	Flu			8.47	5.07	2.18	1.17	313	138	47.12	2.33	143.58	0.54
C094	female	8.3	Flu			7.25	5.98	0.55	0.55	241	123	29.41	10.87	438.18	1
C095	female	9.8	Flu			6.47	4.75	0.95	0.67	271	122	6.64	5	285.26	0.71
C096	male	6.3	Flu			5.14	3.53	0.9	0.44	202	127	2.16	3.92	224.44	0.49
C097	female	5	Flu			7.25	5.26	1.52	0.44	186	118	5.18	3.46	122.37	0.29
C098	male	16	Flu			7.43	5.55	1.1	0.76	161	146	23.45	5.05	146.36	0.69
C099	male	10.6	Flu			4.2	3.17	0.55	0.44	212	133	2.74	5.76	385.45	0.8
C100	male	5.9	Flu			5.63	2.3	2.6	0.59	225	125	5.39	0.88	86.54	0.23
C101	male	11	Flu			6.95	2.63	0.1	3.29	205	142	6.92	26.3	2050	32.9
C102	female	9.3	Flu			5.98	1.97	1.21	2.66	246	138	5.98	1.63	203.31	2.2
C103	female	11.4	Flu			4.02	1.42	1.81	0.53	251	146	26.06	0.78	138.67	0.29
C104	male	12	Flu			7.47	6.01	0.96	0.45	230	152	7.21	6.26	239.58	0.47
A001	male	7.9	Normal			7.77	3.71	3.32	0.6	295	148		1.12	88.86	0.18
A002	male	6.1	Normal			6.39	3.45	2.47	0.31	238	128		1.4	96.36	0.13
A003	female	2.9	Normal			8.47	4.95	2.92	0.41	399	133		1.7	136.64	0.14
A004	male	5.1	Normal			10.32	7.66	1.99	0.57	466	117		3.85	234.17	0.29
A005	male	4.6	Normal			7.22	3.56	3.11	0.35	368	117		1.14	118.33	0.11
A006	female	8.1	Normal			3.55	1.54	1.61	0.33	275	132		0.96	170.81	0.2
A007	male	7.3	Normal			4.97	2.12	2.43	0.29	301	141		0.87	123.87	0.12
A008	female	5.1	Normal			7.03	2.55	4.08	0.18	381	126		0.63	93.38	0.04
A009	male	7.1	Normal			5.11	2.49	2.16	0.31	243	122		1.15	112.5	0.14
A010	male	3.3	Normal			7.1	3.15	3.1	0.51	425	132		1.02	137.1	0.16

A011	male	4.2	Normal			8.34	4.01	3.52	0.5	369	122		1.14	104.83	0.14
A012	female	4.5	Normal			6.88	3.55	3.05	0.44	394	129		1.16	129.18	0.14
A013	female	4.9	Normal			5.38	2.82	2.13	0.31	413	125		1.32	193.9	0.15
A014	male	5.1	Normal			5.36	1.38	3.61	0.19	374	130		0.38	103.6	0.05
A015	female	4.6	Normal			7.48	4.26	2.72	0.34	259	117		1.57	95.22	0.13
A016	female	4.5	Normal			9.46	3.98	4.72	0.62	333	132		0.84	70.55	0.13
A017	female	3.8	Normal			9.4	4.48	4.33	0.47	306	114		1.03	70.67	0.11
A018	female	5.2	Normal			6.38	3.03	2.89	0.33	412	123		1.05	142.56	0.11
A019	female	4	Normal			3.28	1.53	1.32	0.34	281	128		1.16	212.88	0.26
A020	male	9	Normal			5.33	2.86	2.01	0.32	345	127		1.42	171.64	0.16
A021	female	8.9	Normal			8.6	4.02	3.76	0.41	390	128		1.07	103.72	0.11
A022	male	7.1	Normal			6.27	2.91	2.45	0.31	343	130		1.19	140	0.13
A023	male	3.9	Normal			5.62	2.24	2.82	0.49	257	132		0.79	91.13	0.17
A024	female	3.5	Normal			8.16	3.56	4.04	0.39	357	131		0.88	88.37	0.1
A025	male	4.1	Normal			9.17	5.38	2.75	0.62	409	143		1.96	148.73	0.23
A026	male	4.6	Normal			8.33	3.63	4.12	0.42	313	127		0.88	75.97	0.1
A027	female	4	Normal			7.15	2.6	4.07	0.43	288	132		0.64	70.76	0.11
A028	male	5	Normal			8.23	4.27	3.19	0.58	350	123		1.34	109.72	0.18
A029	male	7.3	Normal			9.3	5.93	2.53	0.39	336	124		2.34	132.81	0.15
A030	female	12.3	Normal			3.36	1.51	1.31	0.38	266	138		1.15	203.05	0.29
A031	female	5.4	Normal			7.7	3.64	3.51	0.42	445	129		1.04	126.78	0.12
A032	male	4	Normal			13.67	6.97	5.66	0.81	387	122		1.23	68.37	0.14
A033	male	3	Normal			14.22	9.97	2.73	0.63	490	126		3.65	179.49	0.23
A034	female	13.1	Normal			5.19	2.61	1.71	0.44	280	130		1.53	163.74	0.26
A035	male	3.7	Normal			9.68	4.06	4.69	0.62	363	132		0.87	77.4	0.13
A036	male	3.3	Normal			7.16	3.04	3.56	0.39	394	133		0.85	110.67	0.11
A037	male	7	Normal			17.07	12.07	3.48	0.6	360	132		3.47	103.45	0.17
A038	male	7.1	Normal			7.9	5.52	1.79	0.51	343	131		3.08	191.62	0.28
A039	male	4.6	Normal			8.8	3.75	4.21	0.5	353	114		0.89	83.85	0.12
A040	female	5.5	Normal			5.66	2.32	3.05	0.19	303	131		0.76	99.34	0.06
A041	male	5.1	Normal			7.05	2.99	3.66	0.33	422	127		0.82	115.3	0.09
A042	male	9.5	Normal			7.7	4.45	2.55	0.57	366	134		1.75	143.53	0.22

A043	female	7.6	Normal			8.68	5.06	2.98	0.53	342	133		1.7	114.77	0.18
A044	female	7	Normal			5.47	2.67	2.4	0.2	164	120		1.11	68.33	0.08
A045	male	5.8	Normal			11.25	5.35	4.89	0.75	421	133		1.09	86.09	0.15
A046	male	5.7	Normal			5.43	3.66	1.06	0.41	247	120		3.45	233.02	0.39
A047	female	10	Normal			6.55	3.6	2.35	0.39	408	141		1.53	173.62	0.17
A048	male	5.1	Normal			9.3	3.87	5.02	0.35	334	133		0.77	66.53	0.07
A049	male	8.9	Normal			6.86	3.25	2.99	0.32	344	131		1.09	115.05	0.11
A050	female	3.1	Normal			9.01	3.2	5.34	0.33	252	125		0.6	47.19	0.06
A051	male	5.9	Normal			4.89	1.59	2.48	0.34	245	135		0.64	98.79	0.14
A052	female	3.9	Normal			7.12	3.18	3.17	0.43	639	93		1	201.58	0.14
A053	male	10.8	Normal			4.67	2.3	1.92	0.35	276	124		1.2	143.75	0.18
A054	male	4.8	Normal			7.37	3.04	3.65	0.35	329	122		0.83	90.14	0.1
A055	male	5.6	Normal			7.75	3.71	3.43	0.41	381	133		1.08	111.08	0.12
A056	female	7.8	Normal			8.57	4.11	3.94	0.47	260	129		1.04	65.99	0.12
A057	female	6.9	Normal			4.93	2.22	2.33	0.34	319	120		0.95	136.91	0.15
A058	male	8.3	Normal			5.81	3.3	1.62	0.38	362	134		2.04	223.46	0.23
A059	male	5.4	Normal			6.66	2.97	3.14	0.42	345	132		0.95	109.87	0.13
A060	male	3.7	Normal			10.69	3.05	6.12	0.71	404	122		0.5	66.01	0.12
A061	female	4.6	Normal			8.17	4.5	3.24	0.34	281	133		1.39	86.73	0.1
A062	male	6.2	Normal			7.09	3.49	2.93	0.38	305	124		1.19	104.1	0.13
A063	male	6.2	Normal			12.99	8.33	4.01	0.53	366	126		2.08	91.27	0.13
A064	female	2	Normal			12.22	3.57	7.87	0.6	346	138		0.45	43.96	0.08
A065	male	7.1	Normal			6.84	3.67	2.43	0.63	524	133		1.51	215.64	0.26
A066	male	3.4	Normal			7.18	2.69	4.04	0.24	355	119		0.67	87.87	0.06
A067	female	14	Normal			5.92	3.29	2.04	0.46	297	115		1.61	145.59	0.23
A068	male	9.3	Normal			5.26	3.23	1.5	0.43	383	129		2.15	255.33	0.29
A069	female	6	Normal			8.25	4.96	2.55	0.4	332	128		1.95	130.2	0.16
A070	male	4	Normal			7.59	1.81	5.33	0.34	258	131		0.34	48.41	0.06
A071	male	4	Normal			10.95	6.19	3.71	0.76	410	123		1.67	110.51	0.2
A072	male	4.5	Normal			15.35	10.69	3.234	0.91	393	129		3.31	121.52	0.28
A073	female	4.1	Normal			9.13	4.77	3.62	0.54	338	123		1.32	93.37	0.15
A074	female	5.7	Normal			5.27	2.89	1.89	0.34	290	134		1.53	153.44	0.18

A075	male	4.1	Normal			6.4	2.85	2.87	0.43	287	119		0.99	100	0.15
A076	male	4	Normal			8.07	4.29	3.04	0.43	385	139		1.41	126.64	0.14
A077	male	6.4	Normal			7.63	2.94	3.6	0.85	245	137		0.82	68.06	0.24
A078	male	5	Normal			8.75	4.44	3.61	0.52	327	123		1.23	90.58	0.14
A079	male	4	Normal			9.93	1.74	7.43	0.59	379	139		0.23	51.01	0.08
A080	male	7.4	Normal			6.15	2.64	3.04	0.38	386	135		0.87	126.97	0.13
A081	female	9.3	Normal			10.11	6.92	2.13	0.77	335	134		3.25	157.28	0.36
A082	male	4.1	Normal			5.35	2.46	2.22	0.34	446	115		1.11	200.9	0.15
A083	male	6.5	Normal			10.23	3.85	5.58	0.46	270	138		0.69	48.39	0.08
A084	male	3	Normal			7.85	3.72	3.48	0.49	295	122		1.07	84.77	0.14
A085	female	4.1	Normal			5.52	3.12	1.97	0.32	317	127		1.58	160.91	0.16
A086	female	10	Normal			6.14	3.42	2.2	0.44	337	123		1.55	153.18	0.2
A087	male	6	Normal			9.55	5.19	3.5	0.6	327	128		1.48	93.43	0.17
A088	female	6.5	Normal			5.18	2.25	2.33	0.44	236	124		0.97	101.29	0.19
A089	female	3.9	Normal			9.11	2.69	5.76	0.45	342	125		0.47	59.38	0.08
A090	female	8	Normal			6.67	2.76	3.11	0.29	366	133		0.89	117.68	0.09
A091	male	13.3	Normal			5.05	2.61	1.94	0.34	288	150		1.35	148.45	0.18
A092	male	4	Normal			6.39	2.05	3.78	0.36	328	120		0.54	86.77	0.1
A093	male	4	Normal			6.61	3.48	2.37	0.35	349	124		1.47	147.26	0.15
A094	male	7.4	Normal			5.1	2.89	1.83	0.32	397	128		1.58	216.94	0.17
A095	female	1	Normal			5.98	2.81	2.21	0.39	445	119		1.27	201.36	0.18
A096	female	5.9	Normal			6.94	2.96	3.52	0.4	472	110		0.84	134.09	0.11
A097	female	8.2	Normal			11.96	9.73	1.64	0.51	288	134		5.93	175.61	0.31
A098	male	6	Normal			7.01	3.01	2.58	0.45	293	128		1.17	113.57	0.17
A099	female	9.9	Normal			7.54	3.88	2.99	0.41	305	120		1.3	102.01	0.14
A100	female	5.8	Normal			7.96	4.19	3.29	0.37	321	127		1.27	97.57	0.11
A101	female	6	Normal			4.51	2.11	4.78	0.29	274	132		0.44	57.32	0.06
A102	male	13.1	Normal			12.17	8.72	2.63	0.6	265	145		3.32	100.76	0.23
A103	female	5	Normal			8.81	5.58	2.72	0.42	267	141		2.05	98.16	0.15

Table S3: Comparison of unadjusted and age/sex-adjusted differential abundance analyses of bacterial genera between groups.

Genus	MPP vs Normal (unadjusted/adjusted)	Flu vs Normal (unadjusted/adjusted)
**Mycobacterium**	**2.20E-17/4.38E-15**	**3.08E-20/3.08E-18**
**Aeromonas**	**1.58E-16/2.51E-13**	**4.19E-03/9.84E-04**
**Enterobacter**	**3.19E-18/1.49E-19**	**1.28E-03/6.35E-03**
**Streptomyces**	**0.0222/5.61E-03**	**2.36E-03/6.35E-05**
**Brevundimonas**	**8.55E-11/8.43E-09**	**4.19E-03/0.0329**
**Moraxella**	**1.26E-17/1.60E-15**	**2.08E-04/9.93E-08**
**Streptococcus**	**0.0180/6.76E-03**	**2.41E-07/9.31E-05**
**Haemophilus**	**4.46E-16/4.78E-18**	**1.98E-14/2.06E-12**
**Neisseria**	**4.64E-06/1.15E-09**	**5.20E-05/9.08E-06**
**Fusobacterium**	**0.0481/0.0253**	**1.80E-05/8.91R-03**
**Porphyromonas**	**1.10E-03/9.73E-05**	**4.90E-07/1.11E-08**
**Rothia**	**4.79E-03/6.79E-04**	**1.58E-07/7.78E-08**
**Mycoplasma**	**8.70E-27/4.79E-25**	**0.196/0.962**

**Pseudomonas**	**2.25E-09/3.68E-07**	**0.0930/0.791**
**Acinetobacter**	**1.04E-15/3.43E-17**	**0.874/0.908**
**Tannerella**	**0.0122/6.84E-03**	**0.806/0.830**
**Desulfovibrio**	**5.06E-08/3.13E-10**	**0.842/0.962**
**Comamonas**	**3.70E-07/6.05E-05**	**0.874/0.622**
**Flavobacterium**	**3.76E-04/5.61E-05**	**0.847/0.791**
**Dolosigranulum**	**3.73E-03/4.86E-05**	**0.878/0.833**
**Alloprevotella**	**0.0129/5.61E-03**	**0.170/0.253**

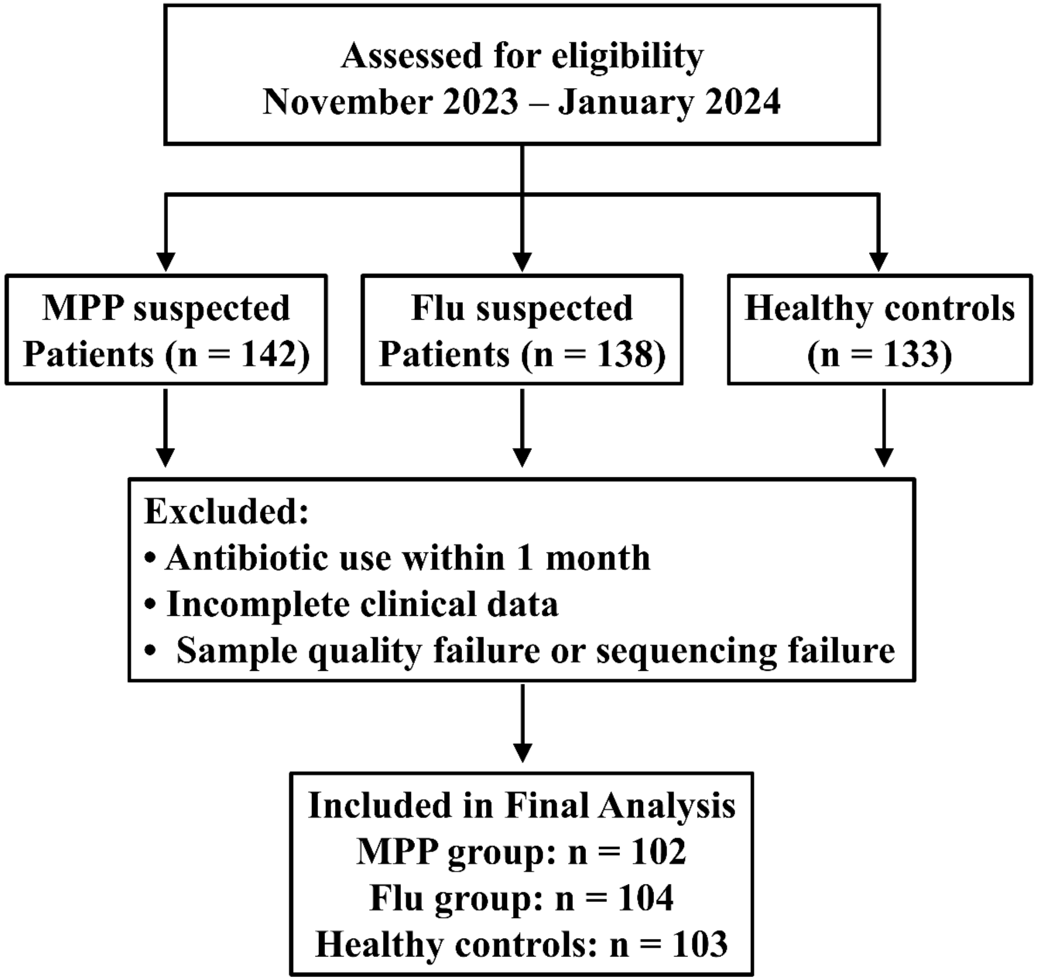

Figure S1: Flow diagram of participant screening, enrollment, and inclusion in the study.

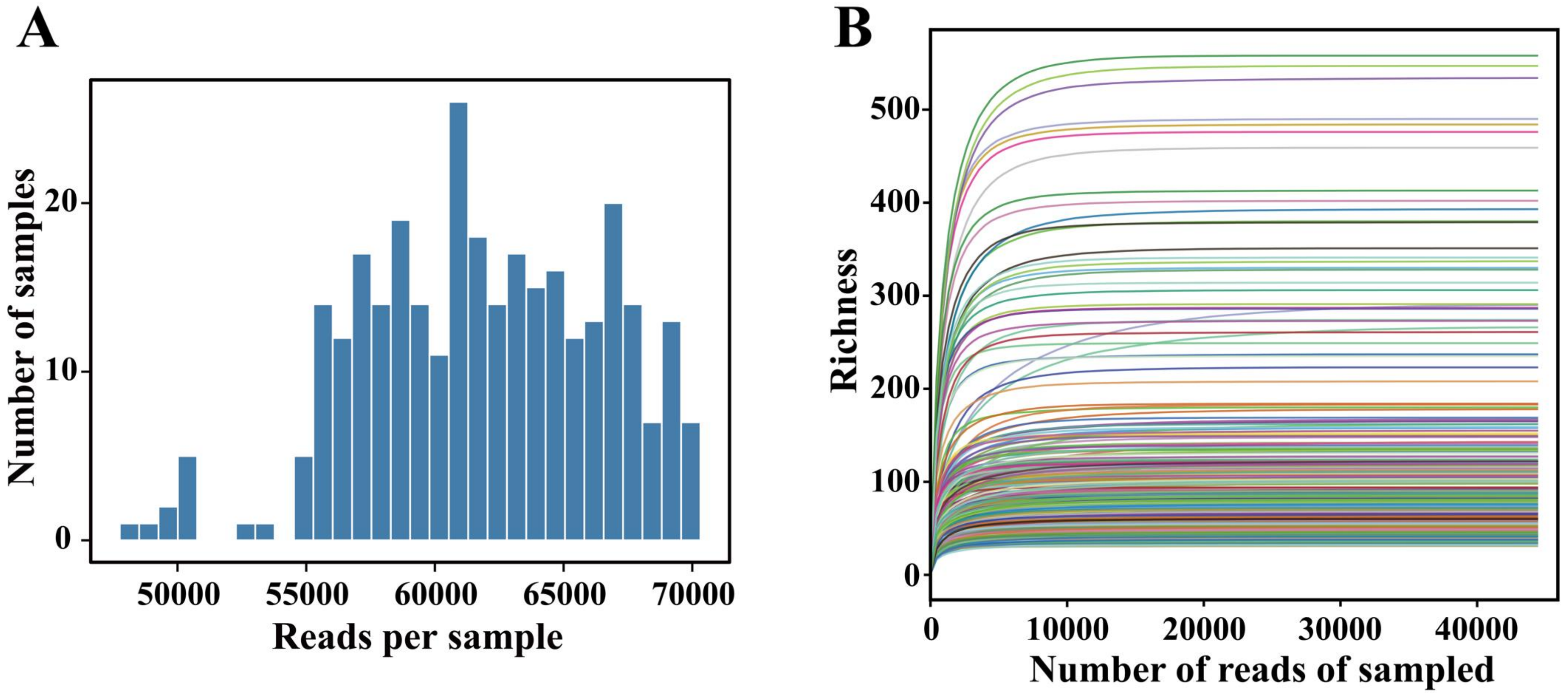

Figure S2: Sequencing depth distribution (A) and rarefaction curves (B) of samples.

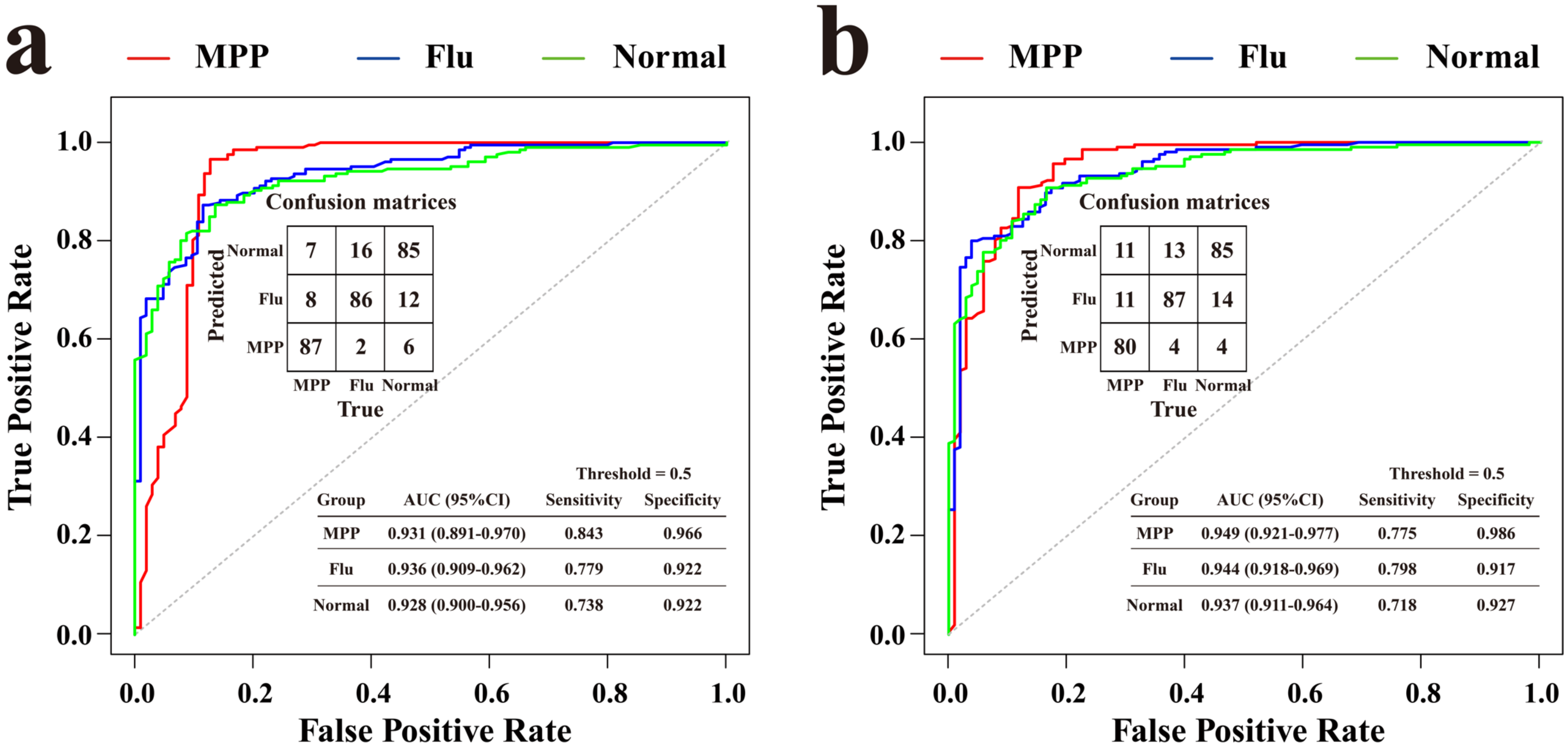

Figure S3: Performance of age-adjusted (a) and *Mycoplasma*-excluded (b) random forest classification models.
